# MHC-*DRB*1/*DQB*1 Gene Polymorphism and Its Association with Resistance/Susceptibility to Cystic Echinococcosis in Chinese Merino Sheep

**DOI:** 10.1155/2014/272601

**Published:** 2014-03-24

**Authors:** Hong Shen, Guohua Han, Bin Jia, Song Jiang, Yingchun Du

**Affiliations:** College of Animal Science and Technology, Shihezi University, Xinjiang, 832003, China

## Abstract

The aim of this study was to analyze the relationship between polymorphism of the MHC-DRB1/DQB1 gene and its resistance to Cystic Echinococcosis (C.E), as well as to screen out the molecular genetic marker of antiechinococcosis in Chinese Merino sheep. The MHCII-DRB1/DQB1 exon 2 was amplified by polymerase chain reaction (PCR) from DNA samples of healthy and hydatidosis sheep. PCR products were characterized by restriction fragment length polymorphism (RFLP) technique. Five restriction enzymes (Mval, HaeIII, SacI, SacII, and Hin1I) were employed to cut DRB1, while seven restriction enzymes (MroxI, ScaI, SacII, NciI, TaqI, Mval, and HaeIII) were employed to cut DQB1.Results showed that frequencies of patterns Mvalbb (*P* < 0.01), SacIab in DRB1 exon 2 (*P* < 0.05), and TaqIaa, HaeIIInn (*P* < 0.01) in DQB1 exon 2 were significantly higher in the healthy group compared with the C.E individuals, which implied that there was a strong association between these genotypes and hydatidosis resistance or susceptibility. Chi-square test showed that individuals with the genic haplotype DRB1-SacIab/DRB1-Mvalbb/DQB1-TaqIaa/DQB1-HaeIIInn (*P* < 0.01) were relatively resistant to C.E, while individuals with the genic haplotypes DRB1-Mvalbc/DQB1-Mvalyy/DQB1-TaqIab/DQB1-HaeIIImn (*P* < 0.01) and DRB1-Mvalbb/DQB1-Mvalcc/DQB1-TaqIab/DQB1-HaeIIImn (*P* < 0.01) were more susceptible to C.E. In addition, to confirm these results, a fielding experiment was performed with Chinese Merino sheep which were artificially infected with *E.g*. The result was in accordance with the results of the first study. In conclusion, MHC-DRB1/DQB1 exon 2 plays an important role as resistant to C.E in Chinese Merino sheep. In addition, the molecular genetic marker of antiechinococcosis (DRB1-SacIab/DRB1-Mvalbb/DQB1-TaqIaa/DQB1-HaeIIInn) was screened out in Chinese Merino sheep.

## 1. Introduction

The major histocompatibility complex (MHC) gene of sheep is located on Chromosome 20 and is called Ovar [1]. The MHC gene family includes two major subfamilies: class I and class II genes [2]. Studies have shown the existence of class II loci that are homologous to HLA-*DQB* [3–6]. As in other vertebrate species, a high degree of polymorphism is found in the Ovar-*DQB* genes, with most of the polymorphic sites located in exon 2, which encodes the antigen-binding site [7]. Due to its highly polymorphic character, a variety of studies have been applied in many fields. It has been well-reported that alleles of different MHC genes correlate with disease resistance in sheep [8]; furthermore, specific MHC alleles are associated with parasite resistance in sheep [9]. Currently, relevant research on Ovar polymorphism and disease resistance or susceptibility mainly concentrates on Ovar-DRB1 [10–14] and Ovar-DQB [7, 15].

C.E is a cosmopolitan zoonotic parasitic disease caused by the larval stage (metacestode stage) of the tapeworm Echinococcus granulosus that cycles between canines, particularly dogs, as definitive hosts and various herbivores as intermediate hosts. In the intermediate hosts and humans, larvae develop into hydatid cysts in various organs, particularly the liver and lungs. C.E is associated with severe morbidity and disability, especially in pastoral areas in northwestern China, the prevalence of which not only results in a considerable decrease in livestock production, but also seriously affects the life quality of people. Chinese Merino sheep, well known as the character of well wool, is beneficial to local sheep husbandary; however it is relatively more susceptible to C.E. Therefore, this disease will result in low performance on Chinese Merino sheep.

At present, many studies focus on MHC-hydatid disease associations in human [16–19]. However, few reports have been published on the study of the Ovar association with C.E in sheep. In this study, efforts were made to investigate MHC-*DRB*1/*DQB*1 gene polymorphism and its association with resistance/susceptibility to C.E in Chinese Merino sheep, screening out the molecular genetic marker of antiechinococcosis.

## 2. Materials and Methods

### 2.1. Animal Sampling and Sample Preparation

We received blood samples from 204 2-year-old Chinese Merino sheep, donated from Mission 165, agricultural division 9, Xinjiang Production and Construction Corps. The C.E sheep and healthy sheep were distinguished by ovine hydatidosis ELISA kit (Shenzhen Combined Biotech Co., Ltd.). We chose 101 C.E sheep and 103 healthy controls. Samples of genomic DNA were obtained from whole blood and stored at −20°C until analysis. The major materials and reagents were obtained from Promega Company and Shanghai Sangon Biological Engineering Technology and Service Co., Ltd.

### 2.2. PCR Amplifications

The second exon of Ovar-*DRB*1 was amplified by nested PCR. The first round of PCR was performed with primers OLA-ERB1 (GC) 5′-CCG GAA TTC CCG TCT CTG CAG CAC ATT TCT T-3′ and HL031 5′-TTT AAA TTC GCG CTC ACCTCG CCG CT-3′ [20]. 100 ng of genomic DNA was used as DNA template in a total volume of 20 *μ*L PCR reaction which was composed of 1.5 mM MgCl_2_ and 120 *μ*M dNTPs, to which 0.2 mM of each primer and 1.5 U of* Taq *polymerase were added. Reactions were performed in a thermocycler under the following conditions: one cycle of initial denaturation for 5 min at 94°C followed by 15 cycles of 94°C for 30 s, 50°C for 30 s, and 72°C for 60 s, with final extension at 72°C for 10 min. Three *μ*L of first step PCR was used for the second step PCR by using primers OLA-ERB1(GC) and OLA-XRBI (5′-AGC TCG AGC GCT GCA CAG TGAAAC TC-3′) [20]. The conditions were one cycle for 5 min at 94°C, followed by 30 cycles of 94°C for 30 s, 63°C for 30 s, and 72°C for 60 s with final extension at 72°C for 10 min. The second exon of* DQB*1 was amplified by primers FW: 5′-CCC CGC AGA GGA TTT CGT G-3′ and REV: 5′-ACC TCG CCG CTG CCA GGT-3′ [21]; 150 ng of Genomic DNA was amplified in a total volume of 112 50 *μ*l, including 1.5 mM MgCl2, 100 *μ*M dNTPs, 0.2 mM of each primer, and 2 U of* Taq *polymerase. Reactions were performed in a thermo cycler under the following conditions: one cycle of initial denaturation for 5 min at 94°C, followed by 33 cycles of 94°C for 30 s, 67°C for 30 s, and 72°C for 45 s, with final extension at 72°C for 10 min.

### 2.3. RFLP

The cleavage map typing method and allele nomenclature referred to that of Konnai et al. [20]. Each 10 *μ*L of* DRB*1 PCR product was digested with 5 U of* Sac*I,* Hin*1I,* Hae*III,* Mva*I, and* Sac*II, respectively, in a total volume of 20 *μ*L, including 2 *μ*L 10× buffer. Each 10 *μ*L of* DQB*1 PCR product was digested with 5 U of* Mrox*I,* Sca*I,* Sac*II,* Nci*I,* Taq*I,* Mva*I, and* Hae*III, respectively. Samples were resolved by agarose gel electrophoresis at varying concentrations (Table S1) (see Supplementary Material available online at http://dx.doi.org/10.1155/2014/272601).

### 2.4. Cloning and Sequencing

According to the typing results of restriction digest, the samples 54 and 74 were selected for cloning and sequencing, because the samples were* Hae*IIImm,* Hae*IIInn,* Mva*Iyy, and* Mva*Izz genotype, which are inconsistent with the previous reports [20]. So the amplified PCR products of these samples were cloned into pGEM-T vector, the ligated plasmids was selected by blue-white colony screening, then masculine clone were sent to sequence.

### 2.5. Verification of Artificial Infection with *E.g*

To verify the validity and reliability of the above research results, sixteen 2-year-old Chinese Merino sheep, which were negative by hydatidosis ELISA kit detection, were chosen to conduct the experiment of artificial infection with* E.g*. Eight of the sheep with the haplotype of* DRB*1-*Sac*Iab/*DRB*1-*Mva*Ibb/*DQB*1-*Taq*Iaa/*DQB*1-*Hae*IIInn were taken as the test group, and the other eight sheep with the haplotypes of* DRB*1-*Sac*Iab/*DRB*1-*Mva*Ibc/*DQB*1-*Taq*Iaa/*DQB*1-*Hae*IIInn,* DRB*1-*Sac*Iab/*DRB*1-*Mva*Ibc/*DQB*1-*Taq*Iaa/*DQB*1-*Hae*IIImn,or* DRB*1-*Sac*Iab/*DRB*1-*Mva*Ibb/*DQB*1-*Taq*Iaa/*DQB*1-*Hae*IIImm, which were not associated with hydatidosis resistance or susceptibility, were taken as the control group. Each sheep was fed 10 adult cestodes with fertilized egg proglottis by mouth. These sixteen sheep were bred under the same conditions.

### 2.6. Statistical Analysis

Allelic and genotypic frequencies in C.E-negative and -positive Chinese Merino sheep were analyzed by *t*-test to assess the relationship between different genotypes and C.E significance. The chi-square test was performed to analyze the relationship between the different haplotypes and C.E resistance. The C.E infection rates of the test and control groups were compared by Fisher's exact test after artificial infection with* E.g*.

## 3. Results

### 3.1. PCR Amplification

Ovar-DRB1 exon 2 was amplified by PCR with primers OLA-ERB1, OLA-HL031, and OLA-XRBI; one specific band of 296 bp was observed on 1.5% agarose (Figure S1B). Ovar-DQB1 exon 2 was amplified by PCR with primers FW and REV, and one specific band of 280 bp was observed on 2% agarose (Figure S1B).

### 3.2. PCR-RFLP

**Figure 1:**
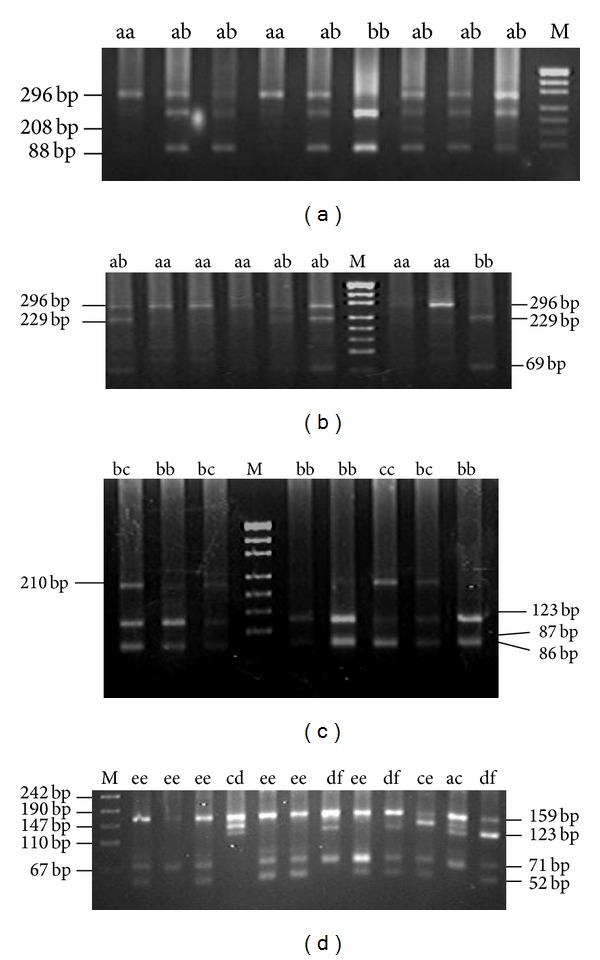
(a) Part results of electrophoretic patterns of exon 2 of MHC-*DRB*1 digested with* Sac*I in Chinese Merino sheep; M: pUC19 DNA marker. (b) Part results of electrophoretic patterns of exon 2 of MHC-*DRB*1 digested with* Sac*II in Chinese Merino sheep; M: pUC19 DNA marker. (c) Part results of electrophoretic patterns of exon 2 of MHC-*DRB*1 digested with* Mva*I in Chinese Merino sheep; M: pUC19 DNA marker. (d) Part results of electrophoretic patterns of exon 2 of MHC-*DRB*1 digested with* Hae*III in Chinese Merino sheep; M: pUC19 DNA marker.

**Figure 2:**
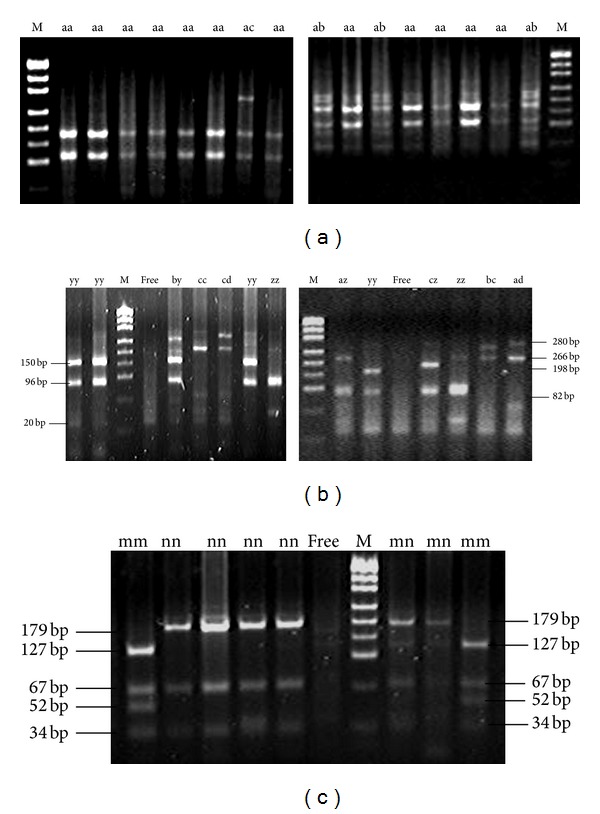
(a) Part results of electrophoretic patterns of exon 2 of MHC-DQB1 digested with TaqI in Chinese Merino sheep; M: pUC19 DNA marker. (b) Part results of electrophoretic patterns of exon 2 of MHC-DQB1. (c) Part results of electrophoretic patterns of exon 2 of MHC-DQB1 digested with* Hae*III in Chinese Merino sheep; M: pUC19 DNA marker.

From restriction digestion of* DRB*1 exon 2 PCR product, genotypes of* Sac*I,* Hin*1I,* Mva*I,* Sac*II, and* Hae*III (Table S2B) were observed, and some of genotypic restriction maps were in Figure 1. In addition, genotypes of restriction enzymes* Mrox*I,* Sca*I,* Sac*II,* Nci*I,* Taq*I,* Mva*I, and* Hae*III (Table S2B) for* DQB*1 PCR products were also observed, and some of their genotypic restriction maps were in Figure 2.

### 3.3. Anlysis of Clonig and Sequncing

We verified the predicted RFLP profiles of Ovar-DRB1 alleles by sequencing cloned 184 amplified products, and all of the observed patterns of fragments matched exactly with those predicted from DNA sequences. Sequencing of Ovar-DQB1 exon 2 cloned amplified products revealed two single point mutations, T to G and A to G, at base positions 32 and 159, respectively, resulting in new alleles,* Hae*IIImm and* Hae*IIInn. In addition, two G-to-A point mutations at base positions 96 and 246 resulted in new alleles,* Mva*Iy and* Mva*Iz. Comparison of sequencing results to the original sequence of DQB1 exon 2 (GenBank, accession numbers: Z28523) are shown in Figure S3.

### 3.4. Analysis of the Relationship between Genotypes and C.E Resistance

Statistical comparisons of genotypic frequencies in C.E sheep and healthy controls revealed that* DRB*1 genotypic frequencies of* Mva*Ibb (*P* < 0.01),* Hae*IIIee, and* Sac*Iab (*P* < 0.05) in negatives were higher than in C.E sheep, indicating a strong association between these genotypes and C.E resistance, while genotypes in terms of* Sac*IIab (*P* < 0.05),* Hae*IIIdf (*P* < 0.05),* Hae*IIIbd (*P* < 0.01), and* Mva*Ibc (*P* < 0.01) in* DRB*1 exon 2 occurred more often in C.E individuals when compared with the healthy group, which implied that there was a strong association between these genotypes and hydatidosis susceptibility (Table 1).* DQB*1 genotypic frequencies of* Taq*Iaa and* Hae*IIInn (*P* < 0.01),* Mva*Idz (*P* < 0.05) in negatives were higher than in positives, while genotypes of* Taq*Iab and* Hae*IIImn (*P* < 0.01),* Mva*Icz (*P* < 0.05) in positives were higher than in negatives (Table 2). Therefore, we concluded that* DQB*1 genotypes of* Taq*Iaa,* Hae*IIInn, and* Mva*Idz were resistant to C.E, while genotypes of* Taq*Iab,* Hae*IIImn, and* Mva*Icz were susceptible to C.E.

### 3.5. Verification of Artificial Infection with *E.g*

Analyzing the haplotype of resistant genotypes, it was found that the haplotype frequency of* DRB*1-*Sac*Iab/*DRB*1-*Mva*Ibb/*DQB*1-*Taq*Iaa/*DQB*1-*Hae*IIInn in C.E-negative sheep was higher than in C.E sheep (*P* < 0.01), indicating that this haplotype was the resistant haplotype of Chinese Merino sheep (Table 3). The result was verified by artificial infection hydatidosis. The haplotypes of* DRB*1-*Mva*Ibc/*DQB*1-*Mva*Iyy/*DQB*1-*Taq*Iab/*DQB*1-*Hae*IIImn and* DRB*1-*Mva*Ibb/*DQB*1-*Mva*Icc/*DQB*1-*Taq*Iab/*DQB*1-*Hae*IIImn in positives were higher than in negatives (*P* < 0.01), which implied that these haplotypes were susceptible to C.E individuals.

Protoscoleces can develop into cysts within 20 days postinfection [22]. The sixteen sheep that were artificially infected with* E.g* were slaughtered in the second month after* E.g* infection, and visual inspection of the liver and lung surfaces of each slaughtered animal was made for the detection of larval stages of cestodes [23]. Results show that 3 sheep were infected with* E.g* in the test group, whereas 6 sheep were infected with* E.g* in the control group; therefore, the infection rate in the test group was significantly lower than that of the control group (*P* < 0.05). It is confirmed that the genic haplotype* DRB*1-SacIab/DRB1-MvaIbb/DQB1-*Taq*Iaa/*DQB*1-*Hae*IIInn leads to C.E resistance in Chinese Merino sheep.

## 4. Discussion and Conclusion

The* MHC* gene is well known to be involved in the vertebrate immune system and encodes antigen recognition proteins used in the adaptive immune response. Polymorphism of this gene has become a hot topic in the past decades. A variety of studies, both overseas and domestic, have shown that MHC of sheep and goats introduces polybase mutation and affluent polymorphism. Amills et al. [24, 25]utilized the PCR-RFLP method to investigate polymorphism of* DRB* in goats, Konnai et al. [20] researched the polymorphism of* DRB*1 in some sheep, with results indicating that affluent polymorphism exists in the Ovis aries-*DRB*1 gene, and Dongxiao and Yuan [26] studied* DRB*3 polymorphism of Chinese local sheep and goats. In addition, Ovis aries-*DQB*1 gene investigations have been conducted abroad [21, 27], and Chinese scholars have studied MHC-*DQ*B and* DQA* in human [28], swine [29], and cattle [30, 31]. However, there are still no domestic reports of Ovis aries-*DQB*1. In the present study, we used* Mrox*I,* Sca*I,* Sac*II,* Nci*I,* Taq*I,* Hae*III, and* Mva*I by PCR-RFLP to analyze* DQB*1 exon 2 and found the existence of 2, 2, 4, 2, 3, 3, and 6 alleles, as well as 3, 3, 7, 3, 4, 6, and 16 genotypes, respectively. The results of cloning and sequencing of the alleles, that is,* Hae*IIIm,* Hae*IIIn,* Mva*Iy, and* Mva*Iz, indicated that they are new alleles resulted from mutation in Chinese Merino sheep.

The extensive diversity at many MHC loci provides a valuable source of genetic markers for examining the complex relationships between host genotype and disease resistance or susceptibility [10]. For example, Sayers et al. [11] suggested that the Ovar-*DRB*1 gene plays an important role in the enhanced resistance of Suffolk sheep to nematode infection. By comparing phenotypic frequencies of A.E patients with healthy controls, it has been speculated that HLA-*DRB*1*11 may have a certain resistance to A.E, but HLA-*DQB*1*02 would exacerbate the disease process [32]. The potential immunogenetic predisposition for susceptibility and resistance to unilocular echinococcosis was investigated by HLA-*DRB*1 typing, and a statistically significant positive association was found between HLA-DR3 and HLA-DR11, and the occurrence of C.E. HLA-DR3 antigen was positively associated with the occurrence of isolated, multiple pulmonary cysts [16]. Differences have been shown between HLA characteristics of A.E patients with different courses of* E.m*, notably the association of the HLA B8, DR3, and DQ2 haplotype with more severe forms of this granulomatous parasitic disease, which suggested that HLA characteristics of the host could influence immune-mediated mechanisms [19]. This study found that the* DRB*1-*Sac*Iab/*DRB*1-*Mva*Ibb/*DQB*1-*TaqI*aa/*DQB*1-*Hae*IIInn haplotype is echinococcosis resistant and selected the genetic markers of resistance to hydatidosis.

In this study, analysis of polymorphisms of MHC-*DRB*1/*DQB*1 by the PCR-RFLP method was performed, as well as screening of genetic markers of antiechinococcosis in Chinese Merino sheep. Artificial infection was used to verify the relationship between different haplotypes of polymorphic MHC gene loci and the resistance of echinococcosis, which would lay a theoretical foundation for sheep breeding of disease resistance in the future.

## Supplementary Material

Fig S1: Electrophoretic patterns of PCR products of exon 2 of Ovar-*DRB*1/DQB1 in Chinese Merino sheep.
Fig S1: 10µl of *DRB*1 exon 2 PCR product was digested with 5U of Hin1I in Chinese Merino sheep, and then Samples were resolved by 1.5% agarose gel electrophoresis.
Fig S2: Each 10µl of *DRB*1 exon 2 PCR product was digested with *5U SacII, MroxI, ScaI, NciI* respectively, The concentration of agarose gel electrophoresis is at2.5%.while concentration of agarose gel concentration of ScaI is 3%.
Fig S3: Comparison of MHC-*DQB*1 sequences of sheep.
TableS1: Conditions of restriction enzymes and examination for PCR products of the exon 2 of MHC-DRB1/ DQB1.
Table S2: The genotypes of PCR-RFLP in exon 2 of the Ovar-*DRB*1/gene.

## Figures and Tables

**Table 1:** Genotypic frequencies of *DRB*1 in Chinese Merino sheep with and without Cystic Echinococcosis.

Cystic Echinococcosis negative			Cystic Echinococcosis positive		
Genotype	Number	Frequency	Genotype	Number	Frequency
*Sac*I aa	42	0.3853	*Sac*I aa	47	0.4700
*Sac*I ab	58	0.5321*	*Sac*I ab	38	0.3800
*Sac*I bb	9	0.0826	*Sac*I bb	15	0.1500
*Hin*1I aa	15	0.1376	*Hin*1I aa	14	0.1414
*Hin*1I ab	55	0.5046	*Hin*1I ab	43	0.4343
*Hin*1I bb	39	0.3578	*Hin*1I bb	42	0.4243
*Sac*II aa	65	0.7471	*Sac*II aa	50	0.6250
*Sac*II ab	13	0.1494	*Sac*II ab	22	0.2750*
*Sac*II bb	9	0.1035	*Sac*II bb	8	0.1000
*Mva*I aa	1	0.0115	*Mva*I aa	0	0
*Mva*I bb	68	0.7816**	*Mva*I bb	45	0.5556
*Mva*I cc	1	0.0115	*Mva*I cc	4	0.0494
*Mva*I dd	0	0	*Mva*I dd	0	0
*Mva*I ab	0	0	*Mva*I ab	0	0
*Mva*I ac	0	0	*Mva*I ac	1	0.0124
*Mva*I bc	17	0.1954	*Mva*I bc	31	0.3827**
*Hae*III aa	17	0.1651	*Hae*III aa	11	0.1100
*Hae*III bb	9	0.0874	*Hae*III bb	4	0.0400
*Hae*III cc	5	0.0485	*Hae*III cc	7	0.0700
*Hae*III dd	0	0	*Hae*III dd	1	0.0100
*Hae*III ee	11	0.1068*	*Hae*III ee	2	0.0200
*Hae*III ff	11	0.1068	*Hae*III ff	8	0.0800
*Hae*III ab	1	0.0097	*Hae*III ab	4	0.0400
*Hae*III ac	12	0.1165	*Hae*III ac	13	0.1300
*Hae*III ae	1	0.0097	*Hae*III ae	4	0.0400
*Hae*III bd	1	0.0097	*Hae*III bd	9	0.0900**
*Hae*III be	2	0.0194	*Hae*III be	5	0.0500
*Hae*III cd	6	0.0583	*Hae*III cd	0	0
*Hae*III ce	16	0.1554	*Hae*III ce	7	0.0700
*Hae*III df	5	0.0485	*Hae*III df	13	0.1300*
*Hae*III ef	6	0.0583	*Hae*III ef	12	0.1200

Note: the same genotypes of *DRB*1 in Chinese Merino sheep with and without Cystic Echinococcosis, *P < 0.05, **P < 0.01.

**Table 2:** Genotypic frequencies of DQB1 in Chinese Merino sheep with and without Cystic Echinococcosis.

Cystic Echinococcosis negative			Cystic Echinococcosis positive		
Genotype	Number	Genotype	Number	Genotype	Number
*Mrox*I aa	31	0.4493	*Mrox*I aa	28	0.4375
*Mrox*I ab	24	0.3478	*Mrox*I ab	25	0.3906
*Mrox*I aa	14	0.2029	*Mrox*I aa	11	0.1719
*Sca*I aa	10	0.1176	*Sca*I aa	14	0.1972
*Sca*I ab	74	0.8706	*Sca*I ab	57	0.8028
*Sca*I bb	1	0.0118	*Sca*I bb	0	0
*Nci*I xx	60	0.7058	*Nci*I xx	66	0.7021
*Nci*I gg	16	0.1882	*Nci*I gg	20	0.2127
*Nci*I xg	9	0.1058	*Nci*I xg	8	0.0851
*Sac*II aa	25	0.4464	*Sac*II aa	37	0.5968
*Sac*II bb	3	0.0536	*Sac*II bb	0	0
*Sac*II cc	5	0.0893	*Sac*II cc	7	0.1129
*Sac*II ab	10	0.1759	*Sac*II ab	4	0.0645
*Sac*II ac	12	0.2143	*Sac*II ac	8	0.1290
*Sac*II ad	1	0.0179	*Sac*II ad	5	0.0806
*Sac*II bd	0	0	*Sac*II bd	1	0.0161
*Taq*I aa	85	0.8252**	*Taq*I aa	58	0.55743
*Taq*I bb	1	0.0097	*Taq*I bb	1	0.0099
*Taq*I ab	17	0.1650	*Taq*I ab	41	0.4059**
*Taq*I ac	0	0	*Taq*I ac	1	0.0099
*Mva*I aa	7	0.0737	*Mva*I aa	3	0.0361
*Mva*I bb	0	0	*Mva*I bb	1	0.0120
*Mva*I cc	13	0.1368	*Mva*I cc	18	0.2169
*Mva*I dd	12	0.1263	*Mva*I dd	4	0.0482
*Mva*I zz	22	0.2316	*Mva*I zz	16	0.1928
*Mva*I yy	15	0.1579	*Mva*I yy	14	0.1687
*Mva*I ad	1	0.0105	*Mva*I ad	0	0
*Mva*I az	3	0.0316	*Mva*I az	1	0.0120
*Mva*I bc	0	0	*Mva*I bc	1	0.0120
*Mva*I bd	0	0	*Mva*I bd	1	0.0120
*Mva*I bz	0	0	*Mva*I bz	1	0.0120
*Mva*I by	2	0.0211	*Mva*I by	0	0
*Mva*I cd	3	0.0316	*Mva*I cd	6	0.0723
*Mva*I cz	7	0.0737	*Mva*I cz	14	0.1687*
*Mva*I dz	9	0.0947*	*Mva*I dz	2	0.0241
*Mva*I dy	1	0.0105	*Mva*I dy	1	0.0120
*Hae*III aa	3	0.0312	*Hae*III aa	0	0
*Hae*III mm	24	0.2500	*Hae*III mm	32	0.3299
*Hae*III nn	55	0.5729**	*Hae*III nn	28	0.2887
*Hae*III am	1	0.0104	*Hae*III am	3	0.0309
*Hae*III an	2	0.0208	*Hae*III an	3	0.0309
*Hae*III mn	11	0.1146	*Hae*III mn	31	0.3196**

Note: the same genotypes of *DQB*1 in Chinese Merino sheep with and without Cystic Echinococcosis, *P < 0.05, **P < 0.01.

**Table 3:** Assessment of the relationship between haplotype and Cystic Echinococcosis in Chinese Merino sheep.

Haplotype of MHC	Number of Cystic Echinococcosis positive cases	Number of Cystic Echinococcosis negative cases	*χ* ^2^
*DRB*1-*Sac*Iab/*DRB*1-*Mva*Ibb/*DQB*1-*Taq*Iaa/*DQB*1-*Hae*IIInn	19	1	17.5734**
*DRB*1-*Sac*Iab/*DRB*1-***Mva*Ibc**/*DQB*1-*Taq*Iaa/*DQB*1-*Hae*IIInn	6	6	0.0012
*DRB*1-*Sac*Iab/*DRB*1-***Mva*Ibc**/*DQB*1-*Taq*Iaa/*DQB*1-***Hae*IIImn**	8	3	2.3000
*DRB*1-*Sac*Iab/*DRB*1-*Mva*Ibb/*DQB*1-*Taq*Iaa/*DQB*1-***Hae*IIImm**	14	11	0.3460
*DRB*1-*Mva*Ibc/***DQB*1**-***Mva*Iyy** */DQB*1-*Taq*Iab/*DQB*1-*Hae*IIImn	0	13	14.1600**
***DRB*1-*Mva*Ibb**/***DQB*1**-***Mva*Icc**/*DQB*1-*Taq*Iab/*DQB*1-*Hae*IIImn	1	18	17.1439**
*DRB*1-*Mva*Ibc/***DQB*1**-***Mva*Ibb**/*DQB*1-*Taq*Iab/***DQB*1-*Hae*IIInn**	4	7	0.9280
*DRB*1-*Mva*Ibc/***DQB*1**-***Mva*Icc**/***DQB*1-*Taq*Iaa**/*DQB*1-*Hae*IIImn	0	2	2.0600
***DRB*1-*Mva*Ibb**/*DQB*1-*Mva*Icz/***DQB*1-*Taq*Iaa**/*DQB*1-*Hae*IIImn	1	5	2.8292

Note: *χ* ^2^ > *χ*_0.01,1_^2^ = 6.63, *P* < 0.01. *χ* ^2^ > *χ*_0.05,1_^2^ = 3.84, *P* < 0.05. *χ* ^2^ < *χ*_0.05,1_^2^ = 3.84, *P* > 0.05.

*P < 0.05, **P < 0.01.

## Abbreviations

MHC: Major histocompatibility complex
Ovar: Ovine MHC
OLA: Ovine lymphocyte surface antigen
PCR: Polymerase chain reaction
RFLP: Restriction fragment length polymorphism
C.E: Cystic Echinococcosis
A.E: Alveolar Echinococcosis
*E.g*: * Echinococcus granulosus*
*E.m*: * Echinococcus multilocularis*.
