# Supplementary material for: MHC-DRB1/DQB1 Gene Polymorphism and Its Association with Resistance/Susceptibility to Cystic Echinococcosis in Chinese Merino Sheep
Source: J Parasitol Res. 2014 Mar 24;2014:272601. doi: 10.1155/2014/272601 (PMC3982463; doi:10.1155/2014/272601)
Supplement: Supplementary file 1 — Fig S1: Electrophoretic patterns of PCR products of exon 2 of Ovar-DRB1/DQB1 in Chinese Merino sheep. Fig S1: 10µl of DRB1 exon 2 PCR product was digested with 5U of Hin1I in Chinese Merino sheep, and then Samples were resolved by 1.5% agarose gel electrophoresis. Fig S2: Each 10µl of DRB1 exon 2 PCR product was digested with 5U SacII, MroxI, ScaI, NciI respectively, The concentration of agarose gel electrophoresis is at2.5%.while concentration of agarose gel concentration of ScaI is 3%. Fig S3: Comparison of MHC-DQB1 sequences of sheep. TableS1: Conditions of restriction enzymes and examination for PCR products of the exon 2 of MHC-DRB1/ DQB1. Table S2: The genotypes of PCR-RFLP in exon 2 of the Ovar-DRB1/gene. [file 272601.f1.pdf]

Supplemetary Materials

Fig.S1 Electrophoretic patterns of PCR products of exon 2 of Ovar-*DRB*1/DQB1 in Chinese Merino sheep

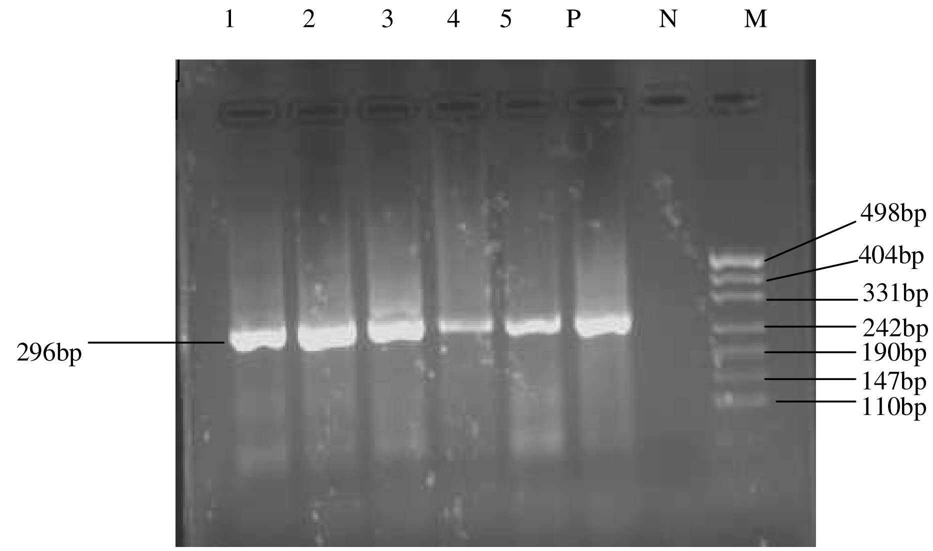

Fig.S1*A*. Electrophoretic patterns of PCR products of exon 2 of Ovar-*DRB*1 in Chinese Merino sheep; M: pUC19 DNA marker. P: positive control N: negative control

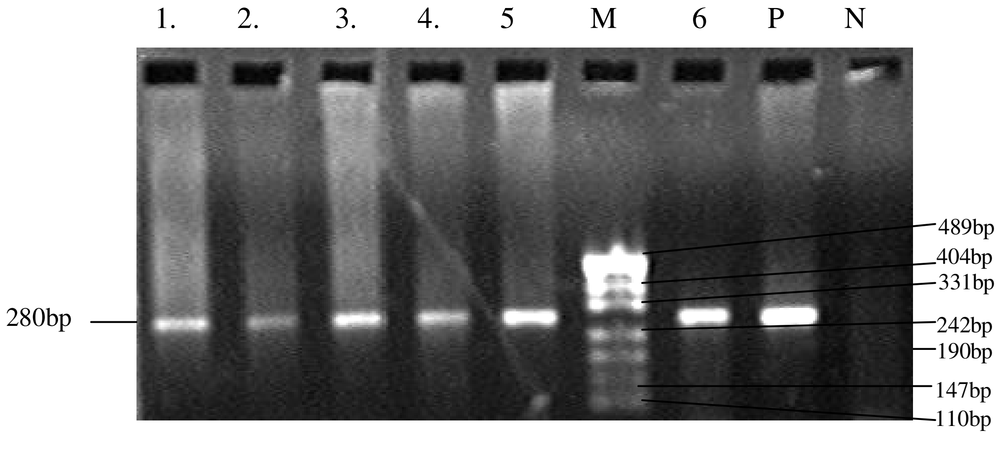

Fig.S1*B*. Electrophoretic patterns of PCR products of exon 2 of *Ovar-DQB*1 in Chinese Merino sheep; M: pUC19 DNA marker. P: positive control; N: negative control

Fig.S1 10μl of *DRB*1 exon 2 PCR product was digested with 5U of *Hin*1I in Chinese Merino sheep, and then Samples were resolved by 1.5% agarose gel electrophoresis

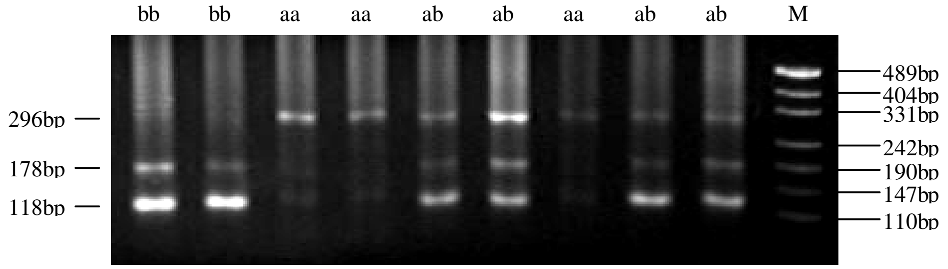

Fig.S1.E. Part results of electrophoretic patterns of exon 2 of MHC-*DRB*1 digested with *Hin*1I in Chinese Merino sheep; M: pUC19 DNA marker.

Fig.S2 each 10μl of *DRB*1 exon 2 PCR product was digested with *5U SacII, MroxI, ScaI, NciI* respectively *, The* concentration of agarose gel electrophoresis is at2.5%.while concentration of agarose gel concentration of ScaI is 3%.

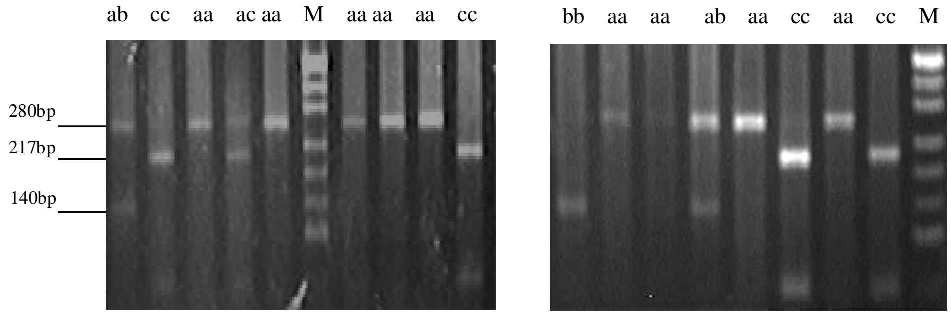

Fig.S2.D Part results of electrophoretic patterns of exon 2 of MHC-*DQB*1 digested with *Sac*II in Chinese Merino sheep; M: pUC19 DNA marker.

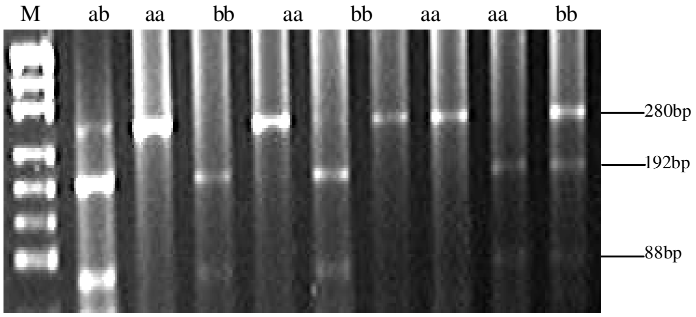

Fig.S2.E Part results of electrophoretic patterns of exon 2 of MHC-*DQB*1 digested with *Mrox*I in Chinese Merino sheep; M: pUC19 DNA marker.

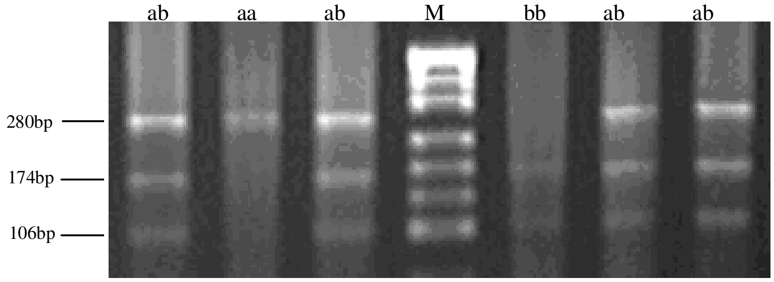

Fig.S2.F. Part results of electrophoretic patterns of exon 2 of MHC-*DQB*1 digested with *Sca*I in Chinese Merino sheep; M: pUC19 DNA marker.

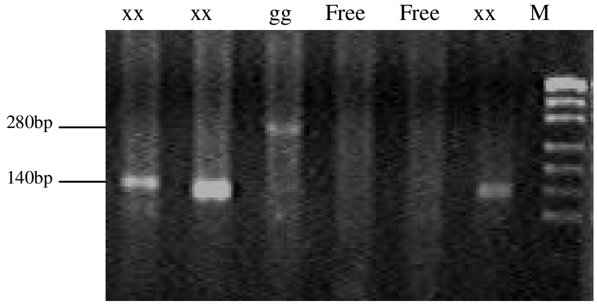

Fig.S2.G. Part reults of electrophoretic patterns of exon 2 of MHC-*DQB*1 digested with *Nci*I in Chinese Merino sheep; M: pUC19 DNA marker.

FigS3. Comparison of MHC-*DQB*1 sequences of sheep.

File: Multiple-Sequence-Alignment

Project:

Number: 3

Maxlength: 273

Names: Ovar-*DQB*1 (origin)   Ovar-*DQB*1-54-1_S   Ovar-*DQB*1-74-1_S

Maxnamelen: 16

Features

Origin

Ovar- *DQB*1 (origin) NATTTCGTGCACCAGTTTAAGTGCCTGTGTTACTTCACCA 40
Ovar-*DQB*1-54-1_S -- n-------------t-------------------t a g-------a c------------------------40
Ovar-*DQB*1-74-1_S -- n-------------t t t----t-----------t - g-------a---------------------------40

Ovar- *DQB*1 (origin) ACGGGACGGAGCGGGTGCGGTACGTGACCAGATACATCTA 80
Ovar-*DQB*1-54-1_S ---c---t---------------------------------80
Ovar-*DQB*1-74-1_S ---a-g-t---------------------------------80

Ovar- *DQB*1 (origin) CAACCGGGAGGAGTACGCGCGCTTCGACAGCGACTGGGAC 120
Ovar-*DQB*1-54-1_S ----------a-----------------t-----t---120
Ovar-*DQB*1-74-1_S ----------a------------------------t---g---120

Ovar- *DQB*1 (origin) GAGTACCGCGGGGTGACGCCGCCGGGGCAGCGGCAAGCCG 160
Ovar-*DQB*1-54-1_S ------c---------g---c-------------c---------t-----------g-----c---g---c--------160
Ovar-*DQB*1-74-1_S ---------------g---c---160

Ovar- *DQB*1 (origin) AGTACTGGAACAGCCAGAAGGACTTCCTGGAGCGGACGCG 200
Ovar-*DQB*1-54-1_S --a---a--------------------------200
Ovar-*DQB*1-74-1_S --a-----------200

Ovar- *DQB*1 (origin) GGCCGAGGTGGACACGGTGTCGAGAAACAACTACCGGGTG 240
Ovar-*DQB*1-54-1_S --g-c--------c------------------a--------240
Ovar-*DQB*1-74-1_S ----------c----------a------------------g-c-------c------------------a--------240

Ovar- *DQB*1 (origin) TATGCCCCCTTCACCTGGCAGCGGCGAGGTNN 272

Ovar-*DQB*1-54-1_S g-a-------g--n 272
Ovar-*DQB*1-74-1_S g-a-------g--n 272

Table.S1. Conditions of restriction enzymes and examination for PCR products of the exon 2 of MHC-*DRB*1/ DQB1

Table.S1*A*. Conditions of restriction enzymes and examination of digested PCR of the exon 2 of MHC-*DRB*1.

Restriction enzymes	Digest- temperature （□）	Digest-time （h）	Concentration of agarose gel（%）	Rcognition site
*Sac*I	37	4	1.5	GAGCT*C
*Hin*1I	37	4	1.5	G（G/A）*CG（C/T）C
*Hae*III	37	4	3	GG*CC
*Sac*II	37	5	2.5	CC GC*GG
*Mva*I	37	8-12	3	CC*（A /T）GG

Note: * represents recognition site; / represents recognition of A or T.

Table.S1*B*. Conditions of restriction enzymes and examination of digested PCR product of exon 2 of MHC-*DQB*1.

Restriction enzymes	Digest- temperature （□）	Digest-time （h）	Concentration of agarose gel（%）	Rcognition site
*Taq*I	65	5	2.5	T*CGA
*Mva*I	37	5	2.5	CC*（A/T）GG
*Hae*III	37	4	2.5	GG*CC
*Mrox*I	37	4	2.5	GAANN*NNTTC
*Sca*I	38	6	3.0	AGT*ACT
*Sac*II	37	4	2.5	CCGC*GG
*Nci*I	37	5	2.5	GG（G /C）*CC

Note: * represents recognition site; / represents recognition of A or T.

Table S2. The genotypes of PCR-RFLP in exon 2 of the Ovar-*DRB*1/gene

Table S2*A*. The genotypes of PCR-RFLP in exon 2 of the Ovar-*DRB*1 gene.

Restriction enzymes	The genotypes of each restriction enzyme			
*Sac*I	aa(296bp)	ab(296bp/208bp/88bp)	bb(208bp/88bp)	
*Hin*1I	aa(296bp)	ab(296bp/178bp/118p)	bb(178bp/118bp)	
*Sac*II	aa(296bp)	ab(296bp/229bp/69bp)	bb(229bp/69bp)	
*Mva*I	aa(296bp)	ac(296bp/210bp/86bp)	bb(123bp/87bp/86bp)	
	cc(210bp/86bp)	bc(210bp/123bp/87bp/86bp)		
*Hae*III	aa(173bp/71bp/48bp/4bp)	bb(173bp/123bp)	cc(159bp/137bp)	dd(159bp/123bp/14bp)
	ee(159bp/71bp/66bp)	ff(159bp/71bp/52bp/14bp)	ab(173bp/123bp/71bp/48bp/4bp)	ac(173bp/159bp/137bp/71bp/48bp/4bp)
	ae(173bp/159bp/71bp/66bp/48bp/4bp)	bd(173bp/159bp/123bp/14bp)	be(173bp/159bp/123bp/71bp/66bp)	cd(159bp/137bp/123bp/14bp)
	ce(159bp/137bp/71bp/66bp)	df(159bp/123bp/71bp/52bp/14bp)	ef(159bp/71bp/66bp/52bp/14bp)	

Table.S2*B*. The genotypes of PCR-RFLP in exon 2 of the Ovar-*DQB*1 gene.

Restriction enzymes	The genotypes of each restriction enzyme			
*Taq*I	aa(165bp/115bp)	bb(231bp/49bp)	ab(231bp/165bp/115bp/49bp)	ac(280bp/165bp/115bp)
*Mrox*I	aa(280bp)	bb(192bp/88bp)	ab(280bp/192bp/88bp)	
*Sca*I	aa(280bp)	bb(174bp/106bp)	ab(280bp/174bp/106bp)	
*Nci*I	gg(280bp)	xx(140bp)	xg(280bp/140bp)	
*Sac*II	aa(280bp)	bb(140bp/140bp)	cc(217bp/63bp)	ab(280/140bp/140bp)
	ac(280bp/217bp/63bp)	bd(140bp/77bp/63bp)	ad(280bp/140bp/77bp/63bp)	
*Hae*III	aa(213bp/67bp)	mm(127bp/67bp/52bp/34bp)	nn(179bp/67bp/34bp)	mn(179bp/127bp/67bp/52bp/34bp)
	am(213bp/179bp/67bp/34bp)	an(213bp/179bp/67bp/34bp)		
*Mva*I	aa(198bp/82bp)	bb(266bp/14bp)	cc(198bp/68bp/14bp)	dd(280bp)
	yy(150bp/96bp/20bp/14bp)	zz(102bp/96bp/48bp/20bp/14bp)	ad(280bp/198bp/82bp)	az(198bp/102bp/96bp/82bp/48bp/20bp/14bp)
	bc(266bp/198bp/68bp/14bp)	bd(280bp/266bp/14bp)	by(266bp/150bp/96bp/20bp/14bp)	bz(266bp/102bp/96bp/48bp/20bp/14bp)

	cd(280bp/198bp/68bp/14bp)	cz(198bp/102bp/96bp/68bp/48bp/20bp/14bp)	dy(280bp/150bp/96bp/20bp/14bp)	dz(280bp/102bp/96bp/48bp/20bp/14bp)
